# Compound Danshen dripping pills prevent early diabetic retinopathy: roles of vascular protection and neuroprotection

**DOI:** 10.3389/fphar.2024.1294620

**Published:** 2024-01-22

**Authors:** Xiaoyu Xu, Mengchen Wang, Shuxia Zhang, Jing Wang, Xinxin Li, Xiaohui Ma, Yun Luo, Xiaobo Sun

**Affiliations:** ^1^ Institute of Medicinal Plant Development, Peking Union Medical College and Chinese Academy of Medical Sciences, Beijing, China; ^2^ Diabetes Research Center, Chinese Academy of Medical Sciences, Beijing, China; ^3^ Beijing Key Laboratory of Innovative Drug Discovery of Traditional Chinese Medicine (Natural Medicine) and Translational Medicine, Beijing, China; ^4^ Key Laboratory of Bioactive Substances and Resource Utilization of Chinese Herbal Medicine, Ministry of Education, Beijing, China; ^5^ State Key Laboratory of Core Technology in Innovative Chinese Medicine, Tasly Pharmaceutical Group Co., Ltd., Tianjin, China

**Keywords:** diabetic retinopathy, CDDP, vascular protection, neuroprotection, apoptosis

## Abstract

**Introduction:** Diabetic retinopathy (DR) represents a major cause of adult blindness, and early discovery has led to significant increase in the number of patients with DR. The drugs currently used for treatment, such as ranibizumab, mainly focus on the middle and late periods of DR, and thus do not meet the clinical need. Here, the potential mechanisms by which compound Danshen Dripping Pills (CDDP) might protect against early DR were investigated.

**Methods:** Db/db mice were used to establish a DR model. The initial weights and HbA1c levels of the mice were monitored, and retinal pathology was assessed by hematoxylin-eosin (HE) staining. The vascular permeability of the retina and thickness of each retinal layer were measured, and electroretinogram were performed together with fundus fluorescein angiography and optical coherence tomography. The levels of inflammatory factors were examined in retinal tissue, as well as those of intercellular adhesion molecule 1 (ICAM-1), IL-6, and monocyte chemoattractant protein 1 (MCP-1) in the serum using ELISA. Immunohistochemistry was used to evaluate levels of vascular endothelial growth factor (VEGF), B-cell lymphoma 2 (Bcl-2), and Bclassociated X protein (Bax). Retinal cell injury and apoptosis were examined by TdT-mediated dUTP Nick End Labeling (TUNEL) assays.

**Results:** The data showed that CDDP significantly improved cellular disarrangement. Imaging data indicated that CDDP could reduce vascular permeability and the amplitude of oscillatory potentials (OPs), and restore the thickness of the ganglion cell layer. Moreover, CDDP reduced the expression levels of inflammatory factors in both the retina and serum.

**Conclusion:** These findings strongly suggest that CDDP prevents early DR through vascular and neuroprotection.

## 1 Introduction

Diabetic retinopathy (DR) is a microvascular consequence of both type 1 and type 2 diabetes, and represents the major cause of vision loss in adults. The number of patients diagnosed with DR is increasing continuously (Sabanayagam et al., 2019). The current understanding of the cellular and molecular pathophysiology of DR is that microvascular damage and neuropathy are key factors in its development (Antonetti et al., 2021). Ranibizumab and conbercept are anti-vascular endothelial growth factor (VEGF) drugs that are currently widely used for retinal macular degeneration (Wang et al., 2018), that occurs in late-stage DR. The use of calcium dobesilate (CaD) to protect the vasculature in DR has also been considered (Zhang et al., 2015). However, there are currently no effective treatments that specifically target early-stage DR. Thus, current treatments cannot satisfy the clinical need, therefore, necessary to find novel approaches for treating early-stage DR.

Compound Danshen dripping pills (CDDP), a patented and well-known traditional Chinese medicine (TCM), are widely used in the emergency treatment of myocardial ischemia in China. Moreover, their safety and efficacy for treating stable angina were demonstrated in a phase III clinical trial (Lei et al., 2014). CDDP is made up of *Salvia miltiorrhiza Bunge* [Lamiaceae; Salviae miltiorrhizae radix et rhizoma], *Panax notoginseng (Burkill) F.H.Chen* [Araliaceae; notoginseng radix et rhizom], and *Cinnamomum camphora (L.) J.Presl* [Lauraceae; borneolum]*,* which is a famous Chinese botanical drug included in Chinese pharmacopoeia (drug code 86900941000087, 27 mg/pill) (Yan et al., 2016), all of which are high in salvianolic acid and ginsenoside (Commission, 2020). Salvianolic acid and ginsenosides have attracted increasing attention due to their protective actions against cardiovascular (Wu et al., 2020) and neurological disorders (Huang et al., 2019). A recent study showed that CDDP could prevent peripheral microvascular impairment brought on by lipid infusion (Zhang et al., 2020) and protect against neuronal damage in rats (Jia et al., 2018). Thus, we speculated that CDDP may have therapeutic effects on DR.

Here, we investigated the protective actions of CDDP against early DR in db/db mice, as well as examining whether the effects are associated with vascular or neuronal protection.

## 2 Material and methods

### 2.1 Reagents

CDDP was gifted by Tianjin Tasly Pharmaceutical Group Co. Ltd. (Tianjin, China). Doxium (calcium dobesilate, CaD) was purchased from Ebewe Pharma Ges. m. b. H. Nfg. KG (Ontara, Austria). Shandong Bausch Lomb Freida Pharmaceutical Co. Ltd provided the carbomer eye drops (Jinan, China). Santen Pharmaceutical (China) Co. Ltd. provided the tropicamide phenylephrine eye drops (Suzhou, China). Fluorescein sodium injection was provided by Alcon Laboratories (Texas, United States). Biosino Biotechnology and Science Inc provided the glycosylated hemoglobin (HbA1c) kits (Beijing, China). Mouse intercellular cell adhesion molecule-1 (ICAM-1), interleukin-1 beta (IL-1β), interleukin-6 (IL-6), and monocyte chemoattractant protein-1 (MCP-1) enzyme-linked immunosorbent assay kits were obtained from Beijing Soleibao Technology Co. Ltd. (Beijing, China). Terminal deoxynucleotidyl transferase-mediated nick-end labeling (TUNEL) and immunohistochemistry kits were purchased from Beyotime Biotechnology Co. LTD (Shanghai, China). The Bax (ab32503) and Bcl-2 (ab182858) primary antibodies were obtained from Abcam (Cambridge, United Kingdom). The remainder of the reagents were bought from Sigma-Aldrich (St Louis, MO, United States).

### 2.2 CDDP preparation and administration

CDDP was prepared by Tianjin Tasly Pharmaceutical Group (Supplementary Table S1). Briefly, *Salvia miltiorrhiza Bunge* and *Panax notoginseng (Burkill) F.H.Chen* were decocted with water and filtered, and the filtrate was concentrated. Add ethanol, leave it to stand and take the supernatant to concentrate. Take appropriate amount of polyethylene glycol and melt it with heat and add the concentrated paste obtained above and ground *Cinnamomum camphora (L.) J.Presl*, drop it into cooled liquid paraffin to get to the Dripping Pills. Separation was performed on Waters Acquity UPLC™ HSS T3 column (2.1 mm × 100 mm, 1.8 μm). The mobile phase was 80% acetonitrile solution containing 0.02% phosphoric acid (A) and 0.02% phosphoric acid (B). The elution gradient was 0–1.6 min, 9%–22% A, 91%–78% B; 1.6–1.8 min, 22%–26% A, 78%–74% B; 1.8–8.0 min, 26%–39% A, 74%–61% B; 8.0–8.4 min, 39%–9% A, 61%–91% B; 8.4–10.0 min, 9% A, 91% B; the flow rate was 0.4 mL/min. The CDDP standard used in this study complied with the Pharmacopoeia of the people’s Republic of China (2020 edition) and was analyzed for quality control of CDDP, and the relevant fingerprints are shown in Supplementary Figure S2.

### 2.3 Animals

**FIGURE 1:**
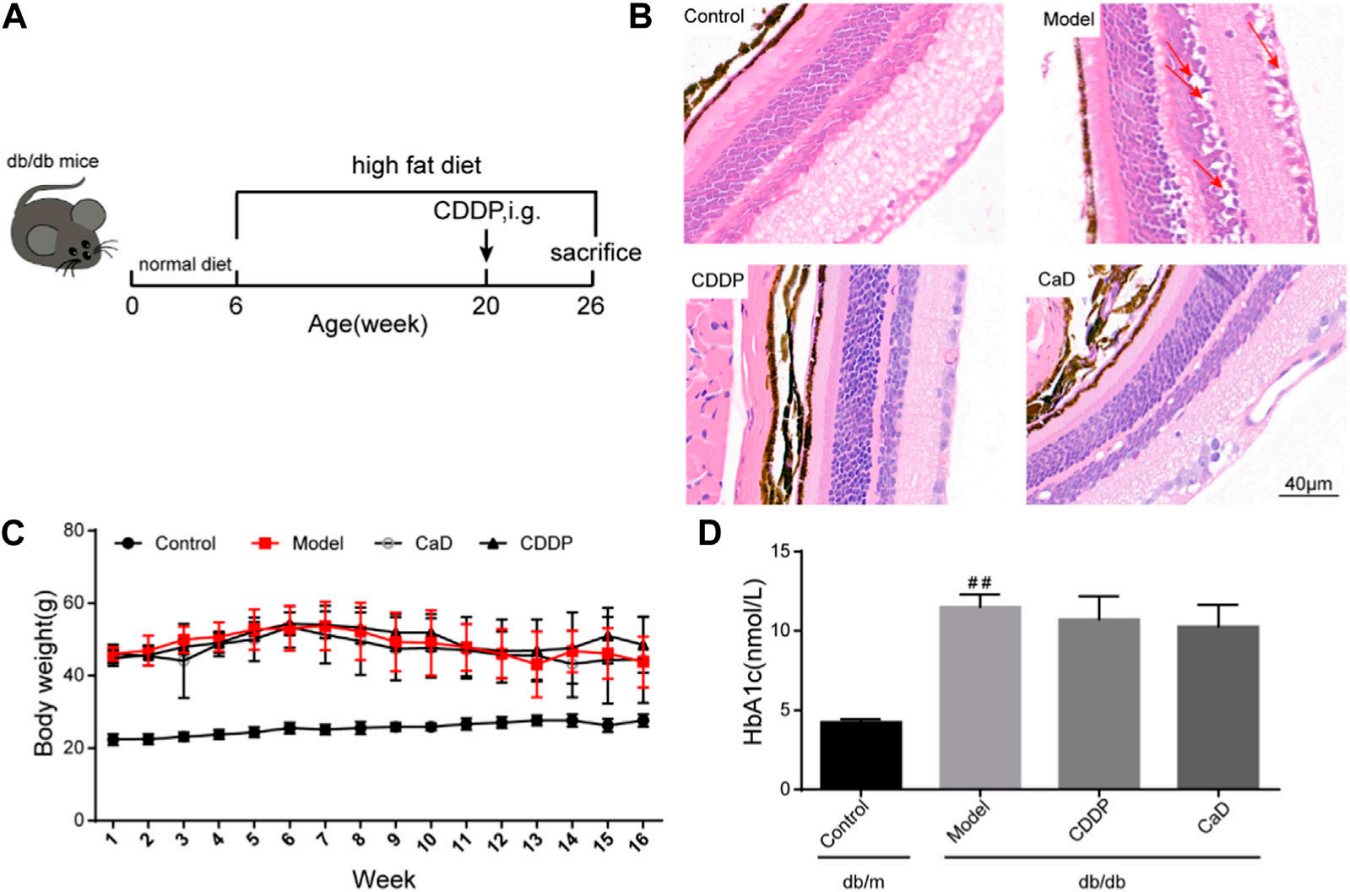
CDDP ameliorates diabetic retinopathy in db/db mice. **(A)** Flow diagram of experiment. **(B)** Representative images of retinal HE staining disordered cells and edema (red arrows). **(C)** Body weight from 1 to 16 weeks in each group. **(D)** HbA1c levels in each group at the end of the experiment. After being normalized to the control (*n* = 6/group), the data is shown as the mean ± SD. ^##^ *p* < 0.01 vs. the Control group.

GemPharmatech Co., Ltd provided 45 male db/db mice aged 20 weeks and 15 male db/m mice aged 20 weeks (Nanjing, China) and raised in SPF-class laboratory housing at the Institute of Medicinal Plant Development (IMPLAD), Chinese Academy of Medical Sciences. The mice lived in a temperature-controlled (temperature: 22°C ± 1°C) facility and could eat and drink freely. They acclimatized in this facility for 1 week before the study began. The 15 db/m mice were used as the controls (I), and the db/db mice were randomly allocated to three groups, namely, the model (II), CDDP (500 mg/kg, i.g.) (III), and positive control CaD (227.5 mg/kg, i.g.) (IV) groups. The body weights of the mice were measured every week. Groups III and IV received orally administered CDDP and CaD for 6 weeks (Figure 1A), whereas groups I and II received saline.

### 2.4 Ethics approval

All animal experiments were approved by the Experimental Animal Ethics Committee of the Institute of Medicinal Plant Development, Peking Union Medical College, in accordance with the National Guidelines for the Care and Use of Animals (SLXD-20190711001).

### 2.5 Glycosylated hemoglobin (HbA1c) assessment

All mice were fasted overnight after CDDP treatment, and HbA1c levels were determined using a detector (SANNUO, China) according to the manufacturer’s recommendations.

### 2.6 Fundus fluorescein angiography (FFA)

Following anesthesia with 1% pentobarbital sodium and 10% sumianxin injection, the mice received tropicamide phenylephrine eye drops to enlarge their pupils and carbomer eye drops to shield them. Fluorescein sodium (10%) was then injected intraperitoneally, and a digital fundus camera was used to identify FFA 2 minutes later (Retinal Imaging System, OptoProbe Research Ltd., Burnaby, Canada). Fluorescent images of retinas were collected and the vessel densities were calculated using AngioTool software (version 1.8.0) according to our latest study (Gao et al., 2020).

### 2.7 Optical coherence tomography (OCT)

Mice were anesthetized with 1% pentobarbital sodium and 10% sumianxin injection. Tropicanamide phenylephrine eye drops were used to dilate the pupils and protected by carbomer eye drops. Mice were then placed on a bench, and their position was fixed according to the head of optic nerve. Retinal OCT was carried out utilizing a retinal imaging system (isOCT, 4D-ISOCT Optoprobe, Burnaby, Canada), and software (Version 2.0) from OptoProbe Research Ltd. was used to determine the thicknesses of the retinal layers.

### 2.8 Electroretinogram (ERG)

Mice were maintained in darkness overnight. The following day, 1% pentobarbital sodium was administered intraperitoneally to mice to make them unconscious and 10% sumianxin in normal saline. Tropicanamide phenylephrine eye drops were used to dilate the pupils and protected by carbomer eye drops. A gold-wire electrode was positioned on the corneal surface of the right eye as a positive pole and another gold-wire electrode was positioned as a negative pole in the flew. The ground was provided via a needle electrode in the tail. The unstimulated left eye was covered with a dark patch. A visual electrophysiological device was used to measure the retinal ERG (OPTO-III, Optoprobe, Burnaby, Canada).

### 2.9 Histopathological analysis

The eyeballs of the mice were harvested, and the retinas were separated under a stereomicroscope. The retinas were dehydrated and embedded in paraffin after being fixed with 4% paraformaldehyde. Paraffin sections (5 µm) were obtained using a Leica RM2235 tissue slicer (Leica, Germany), and were stained using hematoxylin and eosin (HE) as described in our previous study (Luo et al., 2015). A digital slide image scanning method was used to obtain retinal histopathology pictures (Aperio CS2, Leica, Germany).

### 2.10 Enzyme-linked immunosorbent assay (ELISA)

The levels of serum sICAM-1, IL-6, and MCP-1 were determined by ELISA, in accordance with the provided protocols.

### 2.11 Quantitative PCR

The extraction of total RNA, reverse transcription to cDNA, RT-PCR and data analysis were performed as described in our previous study (Luo et al., 2015). The primers were designed using premier primer Software 6.0 (Canadian Premier Life Insurance Company, Toronto, Canada) and the sequences are shown in Table 1.

**TABLE 1:** RT-PCR primers for analysis.

Gene	Direction	Sequence
GAPDH	F	TCTCCTGCGACTTCAACA
	R	TGT​AGC​CGT​ATT​CAT​TGT​CA
IL-6	F	CCA​AGA​GGT​GAG​TGC​TTC​CC
	R	CTG​TTG​TTC​AGA​CTC​TCT​CCC​T
TNF-α	F	CCC​TCA​CAC​TCA​GAT​CAT​CTT​CT
	R	GCT​ACG​ACG​TGG​GCT​ACA​G
IL-1β	F	GCA​ACT​GTT​CCT​GAA​CTC​AAC​T
	R	ATC​TTT​TGG​GGT​CCG​TCA​ACT

### 2.12 Immunohistochemical (IHC) assay

The IHC assay was performed according to the DAB kit instructions. Briefly, 5-μm retinal sections were deparaffinized in xylene and ethanol at various concentrations (100%, 100%, 90%, 80%, and 70%). The sections were then applied for antigen restoration and incubated with primary antibodies at 4 °C for 12 h. Following washing, sections were incubated for 1 h at 37°C with secondary antibodies. DAB was used for visualization and the images were collected using the Leica’s Aperio CS2 digital slide image scanning device, and analyzed by Image Pro Plus (version 6.0; Media Cybernetics, Maryland, United States).

### 2.13 TUNEL assay

Following the manufacturer’s recommendations, the TUNEL assay was used to find retinal cell apoptosis. The TUNEL assay was performed according to our previous study (Gao et al., 2020). Briefly, retinal paraffin sections were deparaffinized, incubated with proteinase K for 30 min and with 3% H_2_O_2_ for 10 min. Sections were then incubated for 1 h at 37°C with a biotin-labeled TUNEL reaction mixture, followed by 30 min at 25°C with streptavidin-HRP working solution. Finally, sections were developed using DAB dye. The number of positive cells was determined after images were captured using a digital slide image scanning system (Aperio CS2, Leica, Germany).

### 2.14 Statistical analysis

Data are presented as mean ±standard error (SEM) using the GraphPad Prism software (version 6.0; San Diego, CA, United States). One-way analysis of variance (ANOVA) and Tukey’s *post hoc* test were used for comparison of multiple groups and *p* < 0.05 was considered statistically significant.

## 3 Results

### 3.1 CDDP ameliorates diabetic retinopathy in db/db mice

To determine whether CDDP influences DR, we first monitored the features of retinal morphological. As shown in Figures 1A, B, the retinas were edematous in the model group with disordered arrangement of the retinal cells, whereas these changes were significantly ameliorated after CDDP treatment. Body weight and blood glucose levels are two fundamental indices of diabetes. As shown in Figures 1C, D, CDDP treatment had no obvious influence on body weight and HbA1c levels, which indicated that the anti-DR effect of CDDP is independent of its hypoglycemic effect.

### 3.2 CDDP mitigates retinal microangiopathy in DR mice

**FIGURE 2:**
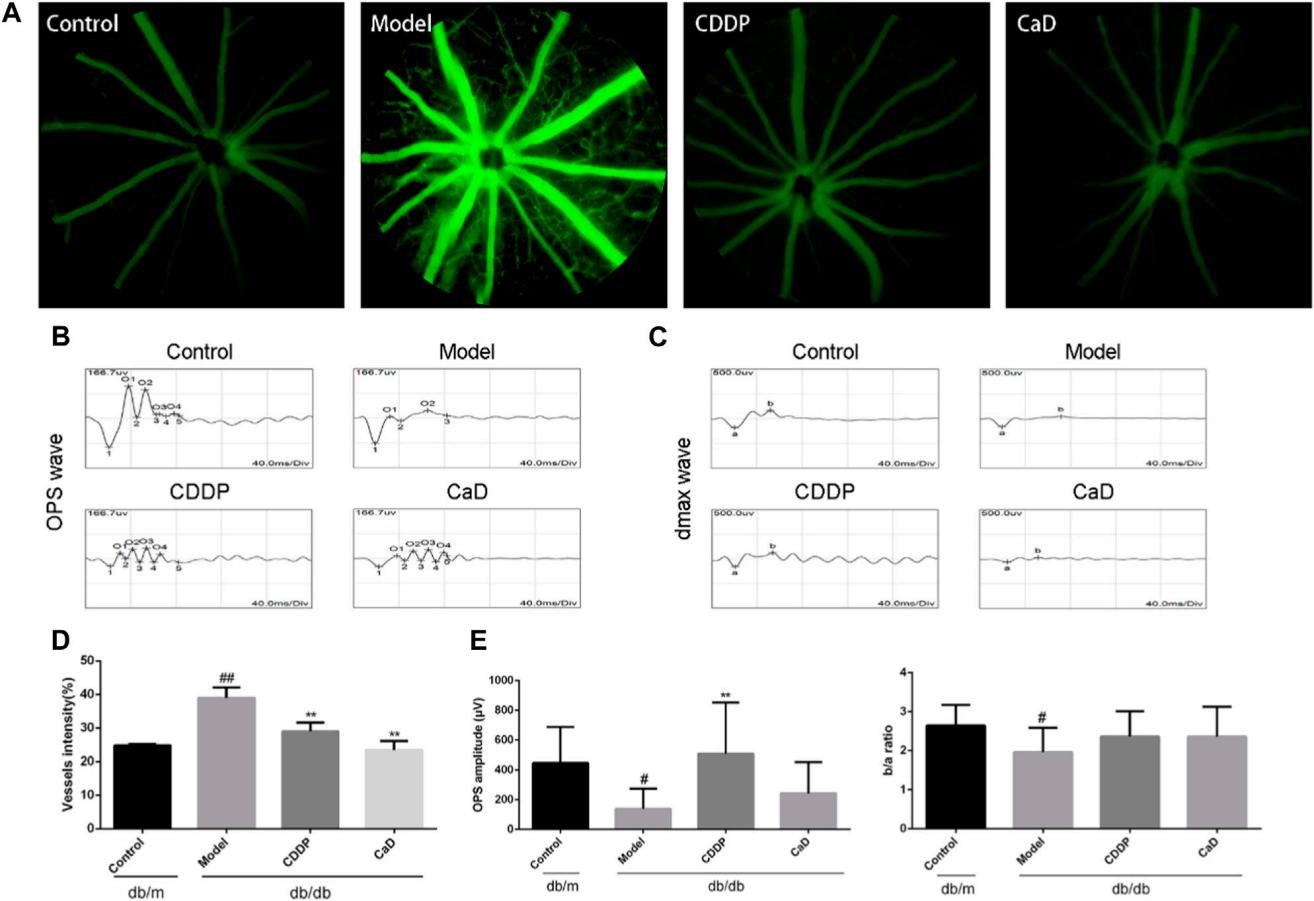
CDDP prevents diabetic microangiopathy in retina of db/db mice. **(A)** Representative images of retinal FFA. **(B)** Representative images of retinal OPS wave. **(C)** Representative images of retinal dmax wave. **(D)** Statistical result of vessels intensity. **(E)** Statistical result of OPs wave and the ratio of b wave to an ERG wave. After being normalized to the control (*n* = 6/group), the data is shown as the mean ± SD. ^#^ *p* < 0.05, ^##^ *p* < 0.01 vs. the Control group; ^**^ *p* < 0.01 vs. the Model group.

Retinal microangiopathy is an early feature of DR; therefore, we evaluated vascular permeability and neovascularization using FFA, and OPs waves by ERG. Figures 2A, D show that there were increased numbers of vessels mice in the DR model group compared to the control group. However, the vessel numbers were significantly reduced following CDDP treatment relative to the DR model group. Moreover, CDDP markedly increased the OPs wave amplitude but had no effect on the b/a ratio (Figures 2B, C, E). Taken together, these results show that CDDP and CaD both protect against vasculopathy in DR mice.

### 3.3 CDDP counteracts neuropathy in DR mice

**FIGURE 3:**
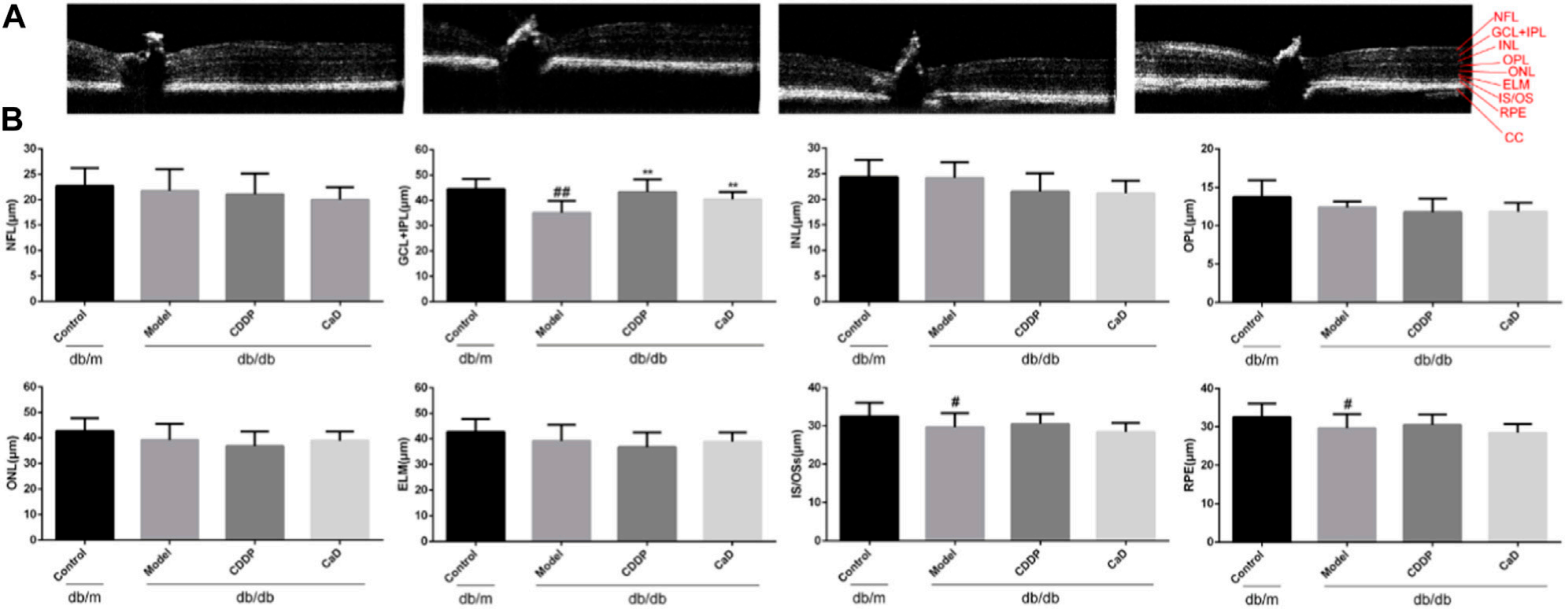
CDDP protects diabetic neuropathy in db/db mice. **(A)** Representative images of retinal OCT. **(B)** Statistical result of NFL, GCL+IPL, INL, OPL, ONL, ELM, IS/OS and RPE layers thickness. The data is presented as the mean ± SD after normalization to the control (n = 6/group). ^#^ *p* < 0.05, ^##^ *p* < 0.01 vs. the Control group; ^**^ *p* < 0.01 vs. the Model group. RPE, retinal pigment epithelium; NFL, nerve fibre layer; GCL, ganglion cell layer; OPL, outer plexiform layer; ONL, outer nuclear layer; IPL, inner plexiform layer; INL, inner nuclear layer; ELM, external limiting membrane; IS/OS, inner and outer segments of photosensitive cells; CC: choroidal capillary.

Retinal neuropathy is another early feature of DR; thus, we analyzed the retinal thickness of each retinal layer using OCT. As depicted in Figures 3A, B, the retinal GCL + IPL, IS/OS, and RPE layers in the model group were markedly reduced in comparison with the controls. However, following CDDP treatment, the thickness of the GCL+IPL layer was markedly increased, while no obvious effects were seen on the IS/OS and RPE layers. This suggests that CDDP protects against neuropathy in mice with early DR.

### 3.4 CDDP reduces the levels of inflammatory factors in DR mice

**FIGURE 4:**
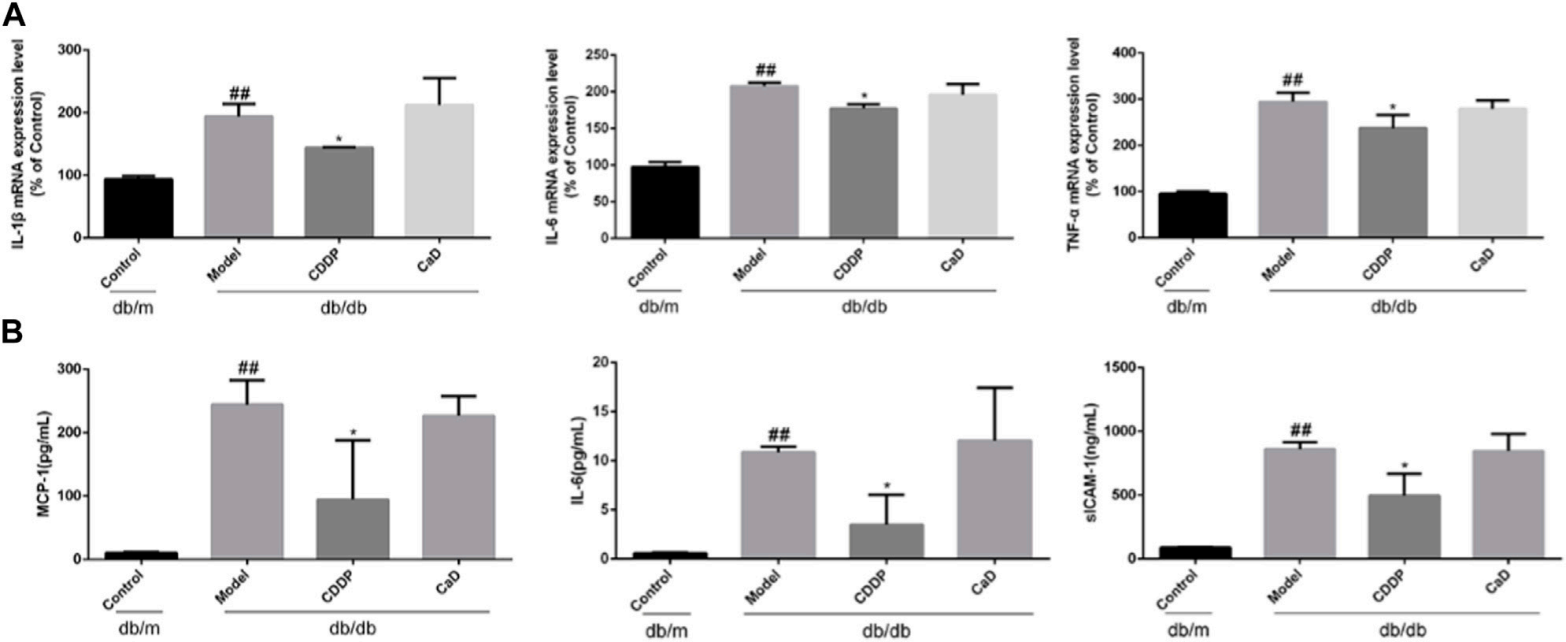
CDDP inhibits serum inflammatory factors expression in db/db mice. **(A)** mRNA levels of retinal tissue detected by qRT-PCR. **(B)** ELISA results of MCP-1, IL-6 and sICAM-1. After being normalized to the control (*n* = 6/group), the data is shown as the mean ± SD. ^##^ *p* < 0.01 vs. the Control group; ^*^ *p* < 0.05, ^**^ *p* < 0.01 vs. the Model group.

Inflammation is a key factor in DR pathogenesis (Forrester et al., 2020). Therefore, we measured the expression of inflammatory factors. CDDP markedly reduced the levels of IL-1β, IL-6, and TNF-α in retinal tissue (Figure 4A), accompanied by reduced expression of MCP-1, IL-6 and sICAM-1 in the sera (Figure 4B), suggesting that CDDP exerted its anti-DR effects by counteracting inflammation.

### 3.5 CDDP inhibits VEGF expression in DR mice

**FIGURE 5:**
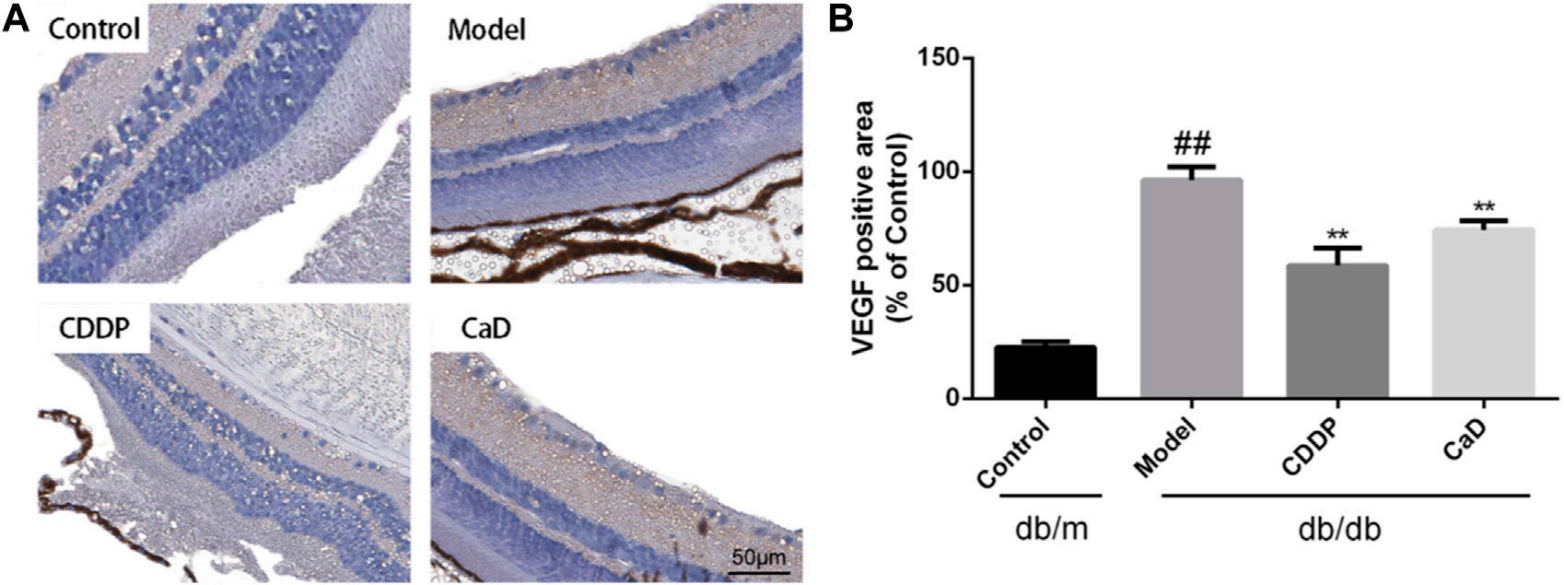
CDDP decreases VEGF expression of retina in db/db mice. **(A)** Representative images of retinal VEGF expression. **(B)** Statistical result of VEGF positive area. After being normalized to the control (n = 6/group), the data is shown as the mean ± SD. ^##^ *p* < 0.01 vs. the Control group; ^*^ *p* < 0.05, ^**^ *p* < 0.01 vs. the Model group.

Excessive VEGF causes pathological vascular permeability and neovascularization, both of which are characteristic of DR (Campochiaro and Akhlaq, 2021). As indicated in Figures 5A, B, CDDP significantly inhibited VEGF expression in the retinas of DR mice.

### 3.6 CDDP prevents retinal cell apoptosis in DR mice

**FIGURE 6:**
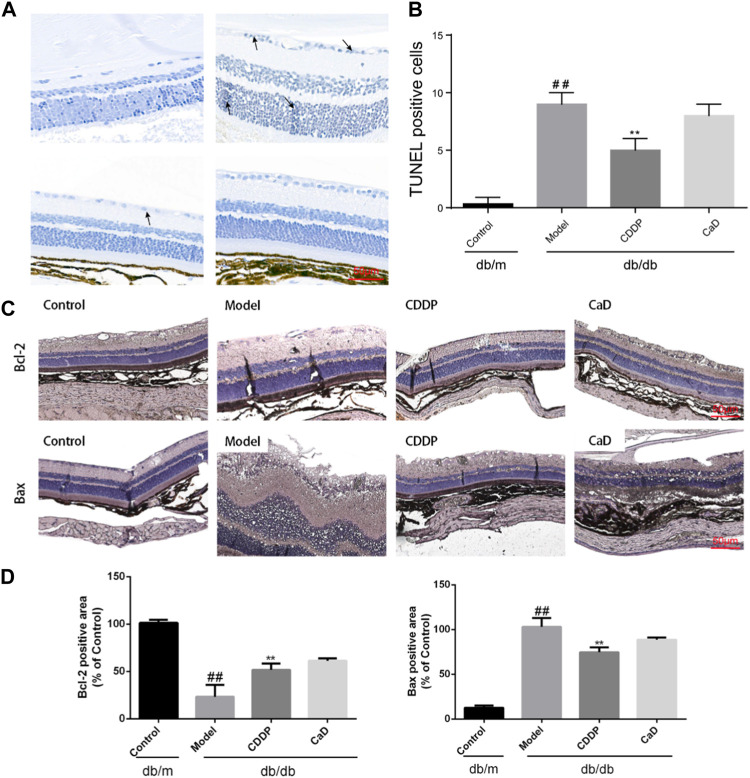
CDDP relieves apoptosis in retinas from db/db mice. **(A)** Representative images of TUNEL staining. **(B)** Statistical result of TUNEL-positive cells. **(C)** Representative IHC images of Bcl-2 and Bax. **(D)** After being normalized to the control (*n* = 6/group), the data is shown as the mean ± SD. ^##^ *p* < 0.01 vs*.* the Control group; ^*^ *p* < 0.05, ^**^ *p* < 0.01 vs*.* the Model gr.

Retinal cell apoptosis is an early feature of DR and was thus evaluated. Figures 6A, B indicate that relative to the controls, increased number of TUNEL-positive cells were observed in the model group, which was reduced following CDDP treatment. And as shown in Figures 6C, D, increased levels of Bcl-2 together with reduced Bax were further indications of the anti-apoptotic effects of CDDP.

## 4 Discussion

DR severely affects the daily lives of people, especially the elderly. Unfortunately, there are no approved drugs for the treatment of early-stage DR; the only available drugs are ranibizumab and aflibercept for treating more advanced DR. Therefore, attention should be given to the development of drugs that can be used for treating early-stage DR. CDDP has been widely used for emergency myocardial ischemia in China since its entry into the market in 1994. Recent randomized, double-blind, dose-parallel, controlled, multicenter clinical trials have revealed additional therapeutic effects of CDDP on DR. In these trials, no significant side effects were observed during the 24-week administration of the drug, in addition to which the “response rate” in the CDDP group was markedly greater than that in the placebo group (Lian et al., 2015). Previous pharmacological studies of CDDP have shown that it can ameliorate DR in rats, together with reducing the apoptosis of retinal cells and thus mitigating DR (Zhang et al., 2018; Liu et al., 2022). However, DR is associated with both microvascular and nerve injuries (Cheung et al., 2010; Simó et al., 2018). The current study is the first time to systematically evaluate the protective action of CDDP on the retina. Furthermore, a more detailed investigation was conducted, comparing the DR-induced damage in different layers of the retina, finding that CDDP was strongly protective against both microvascular and neuronal damage in early-stage DR. Thus, we investigated how CDDP protects against early DR in db/db mice, as well as its mode of action.

Blood glucose levels and weight are the two basic indices used in DR therapy. These were monitored, and the results showed that CDDP had no obvious effect on blood glucose levels. However, retinal histopathology is the gold standard for the detection of DR. Our findings showed that CDDP markedly reduced retinal edema and restored the ordered arrangement of the retinal cells. Taken together, these findings show that the effects of CDDP were independent of both blood glucose and body weight.

Increased retinal vascular permeability and capillary occlusion are typical features of early-stage DR (Wang and Lo, 2018). The FFA results indicated that CDDP reduced the density of vessels, consistent with the findings of our previous study (Gao et al., 2020). Recent studies have shown that lower OPs in the ERG reflect early changes in the microvasculature, characteristic of early-stages DR (Suzumura et al., 2020; Ebihara et al., 2021). Our data demonstrated that CDDP significantly improved the OP amplitude relative to the model group, as previously reported (Yang et al., 2017). VEGF is used as a marker for DR diagnosis and therapy in the clinic and represents an indicator of neovascularization. It was found that CDDP significantly reduced VEGF expression in the retina. Taken together, the results suggest that the anti-DR effects of CDDP may be mediated through protection of the microvasculature.

Retinal neuropathy is also characteristic of early DR, although whether it precedes microvascular changes remains uncertain (Sohn et al., 2016). Moreover, the retinal GCL becomes thinner in DR, suggesting its potential as a therapeutic target (Oshitari, 2021). Currently, topically administered GLP-1 receptor agonists and dipeptidyl peptidase IV (DPP-IV) inhibitors have been reported to have neuroprotective effects on retinal cells, but it is worth mentioning that the therapeutic measures used to ameliorate DR by modulation of the GLP-1-related pathway remain questionable (Hernández et al., 2016). On the one hand, the available experimental evidence is limited and contradictory, and there is also an absence of direct evidence from clinical trials on its therapeutic effects on DR. On the other hand, there are reports showing that GLP-1 analogs such as semaglutide, which are commonly used for treating type 2 diabetes, may promote DR progression (Hernández et al., 2017). Somatostatin and Erythropoietin (EPO) also have some neuroprotective effects (Hernández et al., 2013; Busch et al., 2014), but a randomized, placebo-controlled study failed to show that the neuroprotective drug Somatostatin was effective in preventing or halting microvascular disease, and there may also be some risk that EPO may promote thrombosis and tumor growth (Barbera and Thomas, 2010; Lippi et al., 2010; Simó et al., 2019). Taurourusodeoxycholic acid (TUDCA), neurotophin-4 (NT-4), and citicoline may also have neuroprotective effects in DR, but further experimental and clinical verification is required (Oshitari, 2021). However, CDDP shows significant potential for treating DR, as demonstrated in clinical studies (Lian et al., 2015; Huang et al., 2017; Zhang et al., 2018). Here, the thickness of retinal layers was assessed, indicating that CDDP treatment ameliorated GCL. Hence, CDDP exerts neuroprotective effects in DR.

Inflammation and apoptosis are closely involved in the pathogenesis of early-stage DR, and targeting inflammatory processes may thus be an effective treatment option (Forrester et al., 2020; Xu et al., 2021). As expected, CDDP reduced the levels of IL-1β, IL-6, and TNF-α in retinal tissue as well as MCP-1 and sICAM-1 levels in sera, as previously reported (Qin et al., 2020). The TUNEL results indicated that CDDP reduced the levels of apoptosis in the retinas of db/db mice. To further verify the anti-apoptotic effects of CDDP during early-stage DR, the levels of Bcl-2 and Bax were determined. The immunohistochemical results indicated that CDDP significantly increased expression of Bcl-2 while reducing that of Bax. It is worth noting that positive control CaD treatment has no obvious effect on inflammation and apoptosis. Collectively, these results show that CDDP elicited anti-inflammatory and anti-apoptotic effects in early-stage DR.

In conclusion, CDDP was found to be effective in reducing the symptoms of early-stage DR through mechanisms associated with vascular protection and neuroprotection, rather than regulating blood glucose levels, suggesting the potential of CDDP as a therapeutic drug for early-stage DR. In terms of mechanism, CDDP was found to have strong anti-inflammatory and anti-apoptotic properties. There are also some limitations in our study. Specifically, to study the protective effects of CDDP on the retina, we performed early exploratory experiments, and considering that we conducted a large number of animal experiments, we used a single-dose study to comply with the 4R principle. However, in future studies, we will investigate the mechanisms underlying vasoprotection and neuroprotection in diabetes mellitus in greater depth and we will study the protective effects of CDDP on the retina more comprehensively.

## Data Availability

The original contributions presented in the study are included in the article/Supplementary Material, further inquiries can be directed to the corresponding authors.
