# Supplementary material for: Compound Danshen dripping pills prevent early diabetic retinopathy: roles of vascular protection and neuroprotection
Source: Front Pharmacol. 2024 Jan 22;15:1294620. doi: 10.3389/fphar.2024.1294620 (PMC10839082; doi:10.3389/fphar.2024.1294620)
Supplement: Supplementary file 1 [file Table1.DOCX]

**Table S1** Characteristics of the three botanical drugs in the CDDP

| Chinese name | Pharmacopoeia | Scientific name | Place of origin | Weight |
| --- | --- | --- | --- | --- |
| Danshen | Salviae miltiorrhizae radix et rhizoma | Salvia miltiorrhiza Bunge | Shanxi | 90g |
| Sanqi | notoginseng radix et rhizom | Panax notoginseng (Burkill) F.H.Chen | Yunnan | 17.6g |
| Bingpian | borneolum | Cinnamomum camphora (L.) J.Presl | Wuzhou; Yunnan | 1g |


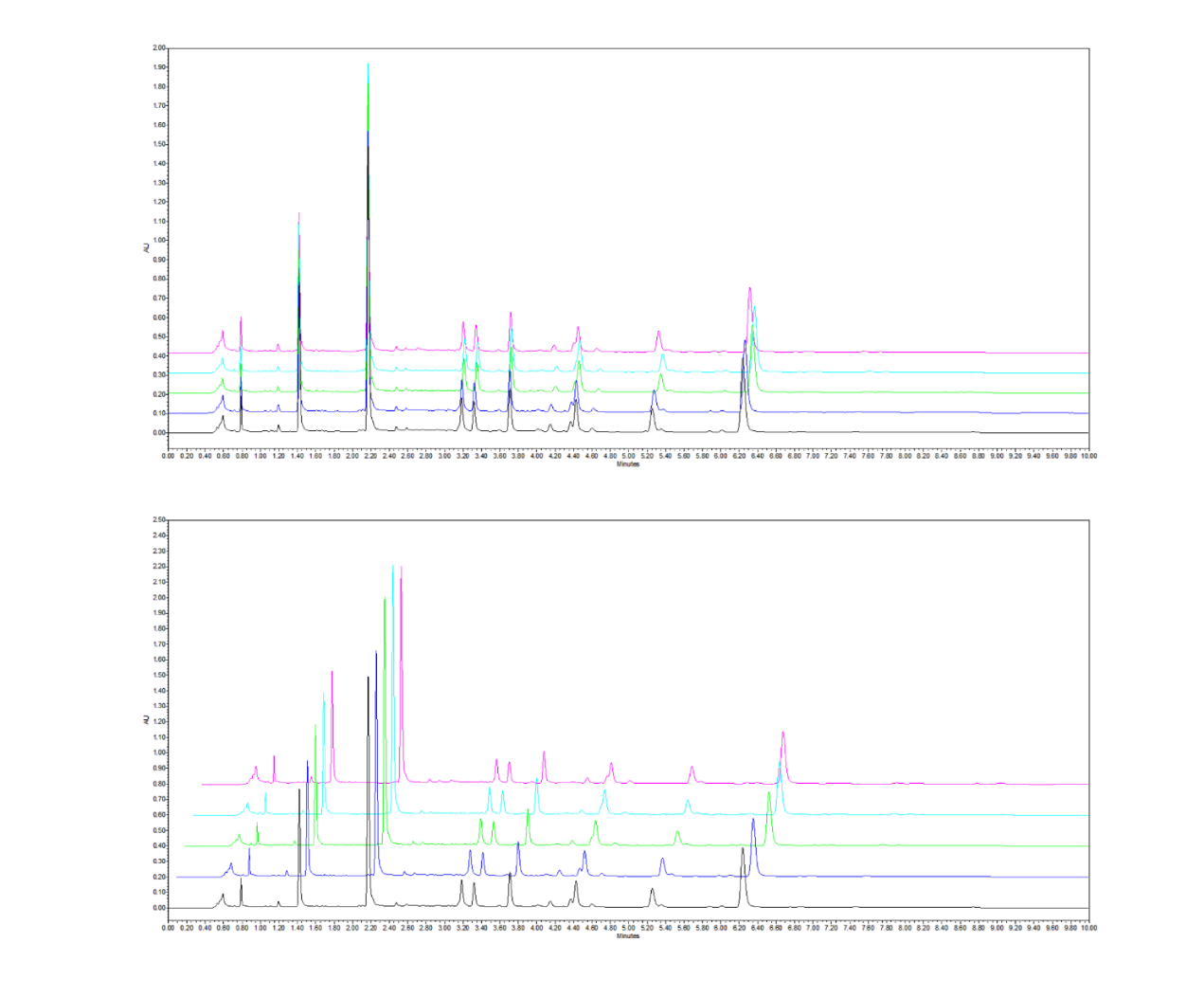


**Supplementary figure** **S2**. UPLC fingerprints of different batches of CDDP at 280nm.
